# Increased lymphoma risk in patients with systemic manifestations of Sjögren’s disease: a population-based study

**DOI:** 10.3389/fmed.2026.1851841

**Published:** 2026-05-29

**Authors:** Teresa Blázquez-Sánchez, Arantxa Torres-Roselló, Javier Llorca, Jorge Mairal-Monesma, Otto Olivas-Vergara, Raquel Largo, Esperanza Naredo, Miguel Ángel González-Gay

**Affiliations:** 1 Department of Rheumatology, Fundacion Jimenez Diaz University Hospital, Madrid, Spain; 2 Joint and Bone Research Unit, IIS-Fundación Jiménez Díaz, UAM, Madrid, Spain; 3 CIBER Epidemiología y Salud Pública (CIBERESP) and Department of Medical and Surgical Sciences, University of Cantabria, Santander, Spain; 4 Department of Medicine and Psychiatry, University of Cantabria, Santander, Spain

**Keywords:** glandular, lymphoma, phenotype, primary Sjögren syndrome, systemic, Sjögren’s disease

## Abstract

**Objective:**

To assess clinical and immunological characteristics, disease activity, and lymphoma development in a well-characterized cohort of patients with Sjögren’s disease, with particular emphasis on differences between glandular and systemic phenotypes.

**Methods:**

We conducted a retrospective observational study of adult patients (≥18 years) with Sjögren’s disease not associated with any other systemic autoimmune rheumatic disease, defined as primary Sjögren’s disease (pSjD), who were seen at a tertiary center in Madrid, Spain. Patients were classified according to the 2016 ACR/EULAR criteria or, alternatively, according to a modified version in which salivary gland ultrasonography (SGUS) was used as an alternative minor criterion when unstimulated whole salivary flow (UWS) and/or ocular staining score (OSS) were not available. For patients diagnosed before 2016, clinical data from medical records were used to apply these criteria retrospectively during the study period. SGUS findings were graded according to the Outcome Measures in Rheumatology (OMERACT) scoring system or based on expert radiologists’ assessment. Systemic disease activity was assessed using the EULAR Sjögren’s Syndrome Disease Activity Index (ESSDAI). Demographic, clinical, laboratory, histopathological, lymphoma occurrence, and treatment data were retrospectively collected. Patients were stratified into glandular (exocrine-limited) or systemic (glandular and systemic involvement) phenotypes.

**Results:**

Among 278 patients included, 148 (53%) had a glandular phenotype and 130 (47%) a systemic phenotype. The mean age at diagnosis was 54.6 (± 14.5 years); 95% were women. Sicca symptoms were highly prevalent in both groups (xerophthalmia 89%, xerostomia 84%). In the systemic group, musculoskeletal involvement was most common, particularly arthritis (38%), followed by cutaneous manifestations (26%), cytopenias (19%), pulmonary involvement (16%), and renal or central nervous involvement (5%). ESSDAI scores were significantly higher in patients with systemic involvement (13.9 ± 21.7 vs. 4.0 ± 7.9; *p* < 0.001). Immunological profiles were similar across phenotypes. Lymphoma occurred in 15 patients (5%), predominantly in patients with systemic involvement (10% vs. 1%; *p* = 0.001), mostly non-Hodgkin lymphoma. Immunosuppressive therapy was more commonly used in patients with systemic involvement.

**Conclusion:**

Adequate stratification of pSjD according to presence or absence of systemic manifestations is mandatory. Our results encourage a close follow-up of systemic pSjD phenotype, particularly for early diagnosis of lymphoma.

## 1 Introduction

Sjögren’s disease is a chronic systemic autoimmune disorder, mainly driven by T and B lymphocytic infiltration of exocrine glands, activation of different signaling pathways and systemic cytokine production, leading to immune-mediated dysfunction of the exocrine glands, particularly the salivary and lacrimal glands, resulting in sicca symptoms, most commonly xerostomia and keratoconjunctivitis sicca (1).

Sjögren’s disease may present as an isolated condition (2) or in association with other autoimmune diseases, such as rheumatoid arthritis and systemic lupus erythematosus (3). When the disease occurs in the absence of another underlying systemic autoimmune rheumatic condition, it can be classified as primary Sjögren’s disease (pSjD).

The interplay of pathogenic mechanisms observed in Sjögren’s disease contributes to the highly heterogeneous clinical expression, which ranges from isolated glandular involvement to multiple systemic manifestations (1, 2). Notably, patients with this disease have an increased risk of developing lymphoma, particularly non-Hodgkin lymphoma (NHL), which represents one of the most severe complications of the disease (1, 4).

Clinically, two main patterns of disease expression are recognized within the spectrum of pSjD: A glandular form, in which manifestations are largely confined to the salivary and lacrimal glands, and a systemic form characterized by involvement of the musculoskeletal, cutaneous, hematological, pulmonary, renal, and neurological systems (5). Distinguishing between these two patterns is clinically relevant, as systemic involvement reflects a higher disease burden and is associated with a worse prognosis, including the development of lymphoma (6). In this context, several clinical, immunological, and biological features have been linked to an increased risk of lymphoma in pSjD, including persistent parotidomegaly, cytopenias, hypocomplementemia, cryoglobulinemia, monoclonal gammopathy, and other markers of B-cell activation (7). However, the available evidence remains heterogeneous, with reported differences in lymphoma risk, histological subtypes, and prognosis across pSjD populations (8–11).

According to the 2016 ACR/EULAR classification criteria, pSjD patients are considered eligible for classification if their total score is 4 or higher based on these items: anti-SSA/Ro positivity and/or focal lymphocytic sialadenitis (focus score ≥ 1 focus/4 mm^2^), each scoring 3 points, and abnormal ocular staining score (OSS), Schirmer’s test ≤ 5 mm/5 min, and unstimulated whole salivary flow (UWS) rate ≤ 0.1 mL/min, each scoring 1 point (12).

Salivary gland ultrasonography (SGUS) is a non-invasive imaging technique that is increasingly used to support the diagnosis of Sjögren’s disease. Ultrasound findings in the major salivary glands show a high level of agreement with histological findings obtained from minor salivary gland biopsy in patients with Sjögren’s disease (13–15). In addition, SGUS of the major salivary glands have demonstrated good reproducibility (16–18) and strong diagnostic performance for this condition (19–21).

The Outcome Measures in Rheumatology (OMERACT) group developed a consensus-based ultrasonographic scoring system to assess semi-quantitative parenchymal changes in the major salivary glands, with scores ranging from 0 to 3 (17, 18, 22). In patients with suspected Sjögren’s disease, an OMERACT score ≥ 2 in at least one major salivary gland is associated with high diagnostic performance for Sjögren’s disease (21). Previous studies to the OMERACT scoring system had already shown that experienced readers could reliably identify the structural changes of Sjögren’s disease with diagnostic value (23–27).

In this context, the incorporation of the SGUS OMERACT score into the ACR/EULAR classification criteria has been shown to preserve the sensitivity and specificity of the current classification system, supporting the integration of SGUS into the diagnostic work-up of patients with suspected Sjögren’s disease (28–30). In addition, some studies have indicated that SGUS may replace minor classification items, including the OSS, Schirmer’s test, or UWS, without substantially altering overall classification performance (30).

Based on these considerations, the objective of this single-center study was to determine whether a well-characterized cohort of pSjD patients, classified according to 2016 ACR/EULAR criteria or modified ACR/EULAR criteria incorporating the SGUS, when necessary, represent clinically distinct glandular and systemic phenotypes with different clinical and immunological characteristics, disease activity burden, and lymphoma occurrence.

## 2 Patients and methods

A retrospective observational study was conducted at the Rheumatology Department of a single tertiary care center in Madrid, Spain (Hospital Universitario Fundación Jiménez Díaz), including patients with Sjögren’s disease not associated with other systemic autoimmune diseases (such as rheumatoid arthritis, systemic lupus erythematosus, or systemic sclerosis), defined as pSjD. For this purpose, we included patients with pSjD aged ≥ 18 years with available clinical, laboratory, histopathological, glandular function, and imaging data.

For the purposes of the present study, patients were classified according to the 2016 ACR/EULAR criteria (12). For patients diagnosed before 2016, clinical data from medical records were used to apply these criteria retrospectively during the study period. Because UWS and OSS were not routinely available at our center, SGUS was used as an alternative minor classification item when available, weighted 1 point and was used to replace unavailable UWS and/or OSS assessments (21, 29–31). Information summarizing the modified classification criteria used for the present study and their corresponding scores is provided in Table 1. Accordingly, patients were considered to fulfill the modified criteria when the total score was ≥ 4.

**TABLE 1:** Modified 2016 ACR/EULAR classification criteria for Sjögren disease used in the study.

Item	Score
Focal lymphocytic sialadenitis in minor salivary gland biopsy with a focus score ≥ 1 focus/4 mm^2^ of glandular tissue	3
Anti-SSA/Ro antibody positivity	3
Major salivary gland ultrasonography positive, defined as at least one major salivary gland with a OMERACT Ultrasound Grey-scale Scoring System of 2 or 3 or based on expert radiologists’ assessment	1
Schirmer’s test ≤ 5 mm/5 min in at least one eye	1
Patients were classified as fulfilling modified criteria when the total score was ≥ 4	

SGUS was used as an alternative minor item weighed 1 point when UWS and/or OSS were unavailable.

Ultrasound examinations were performed either by a rheumatologist with extensive experience in SGUS (EN), who was involved in the development of the OMERACT scoring system (17), or by experienced radiologists at our center.

Systemic disease activity in pSjD was assessed using the EULAR Sjögren’s Syndrome Disease Activity Index (ESSDAI), which allows standardized evaluation of systemic involvement across organ domains, categorized as low (<5), moderate (5–13) or high activity ( ≥ 14) (32).

Exclusion criteria included Sjögren’s disease associated with other well-defined systemic diseases (including rheumatoid arthritis, systemic lupus erythematosus) and patients with insufficient data for disease classification.

The study procedures followed the recommendations of the 1975 Declaration of Helsinki, revised in 2024. It was approved by the Clinical Research Ethics Committee of Fundación Jiménez Díaz, Madrid (Spain) (protocol No. PIC249-24_FJD).

### 2.1 Data collection

Data were retrospectively extracted from medical records. Variables were collected at diagnosis and follow-up when applicable.

Demographic variables included age, gender, ethnicity, age at pSjD diagnosis and disease duration.

Laboratory investigations included anti-SSA/Ro and anti-SSB/La antibodies, antinuclear antibodies (ANA), rheumatoid factor, serum complement levels (C3 and C4), β2-microglobulin levels, serum immunoglobulin levels and the presence of monoclonal gammopathy of undetermined significance.

Histopathological findings of minor salivary gland biopsies were assessed when available. A positive salivary gland biopsy was considered when a focus score ≥ 1 focus per 4 mm^2^ was identified, in accordance with established definitions (12).

All available SGUS data were collected. Examinations performed by EN were scored by her according to the OMERACT scoring system (grades 0–3) (17). Those performed by radiologists were described as consistent with Sjögren’s disease when structural abnormalities of the major salivary glands were described such as parenchymal heterogeneity, focal or diffuse hypoechoic areas, cystic changes or hyperechoic bands, calcifications (21, 23–27).

Neoplastic events were recorded and classified as occurring before or after the diagnosis of pSjD. Lymphoma subtypes (including non-Hodgkin lymphoma) and leukemia were documented when present.

Current and previous treatments were recorded, including hydroxychloroquine, conventional synthetic DMARDs (azathioprine, methotrexate, leflunomide, and mycophenolate mofetil), biologic agents (including rituximab), intravenous immunoglobulins and systemic glucocorticoids.

### 2.2 Group assignment

Patients were classified into two mutually exclusive clinical phenotypes, glandular disease (exocrine-limited) or systemic disease, according to the presence of systemic organ involvement, in line with commonly described domains in pSjD (5).

*Glandular phenotype:* Defined as disease limited to exocrine gland involvement, including xerophthalmia, xerostomia, in the absence of any systemic manifestations.

*Systemic phenotype*: Defined by the presence of at least one manifestation attributed to pSjD beyond the exocrine glands, according to the following organ domains: Articular (arthritis), muscular (myositis), hematological (cytopenias), cutaneous (including leukocytoclastic vasculitis), pulmonary, renal, and neurologic (peripheral or central nervous system).

### 2.3 Statistical analysis

Statistical analyses were performed using the package Stata 19/SE (College Station, TX, United States). We describe continuous variables as mean ± standard deviation, and categorical variables as number and percentage. Comparisons between glandular and patients with systemic involvement were carried out with Student’s *t*-test for continuous variables (or Mann-Whitney U test if needed) and chi-squared or Fisher’s exact test for categorical variables. The association between glandular Sjögren disease and lymphoma was also studied via logistic regression adjusting for current age and duration of the Sjögren disease. Its results are reported as odds ratios (OR) with 95% confidence intervals (CI). *P*-values are reported without correction for multiple comparisons.

## 3 Results

**FIGURE 1:**
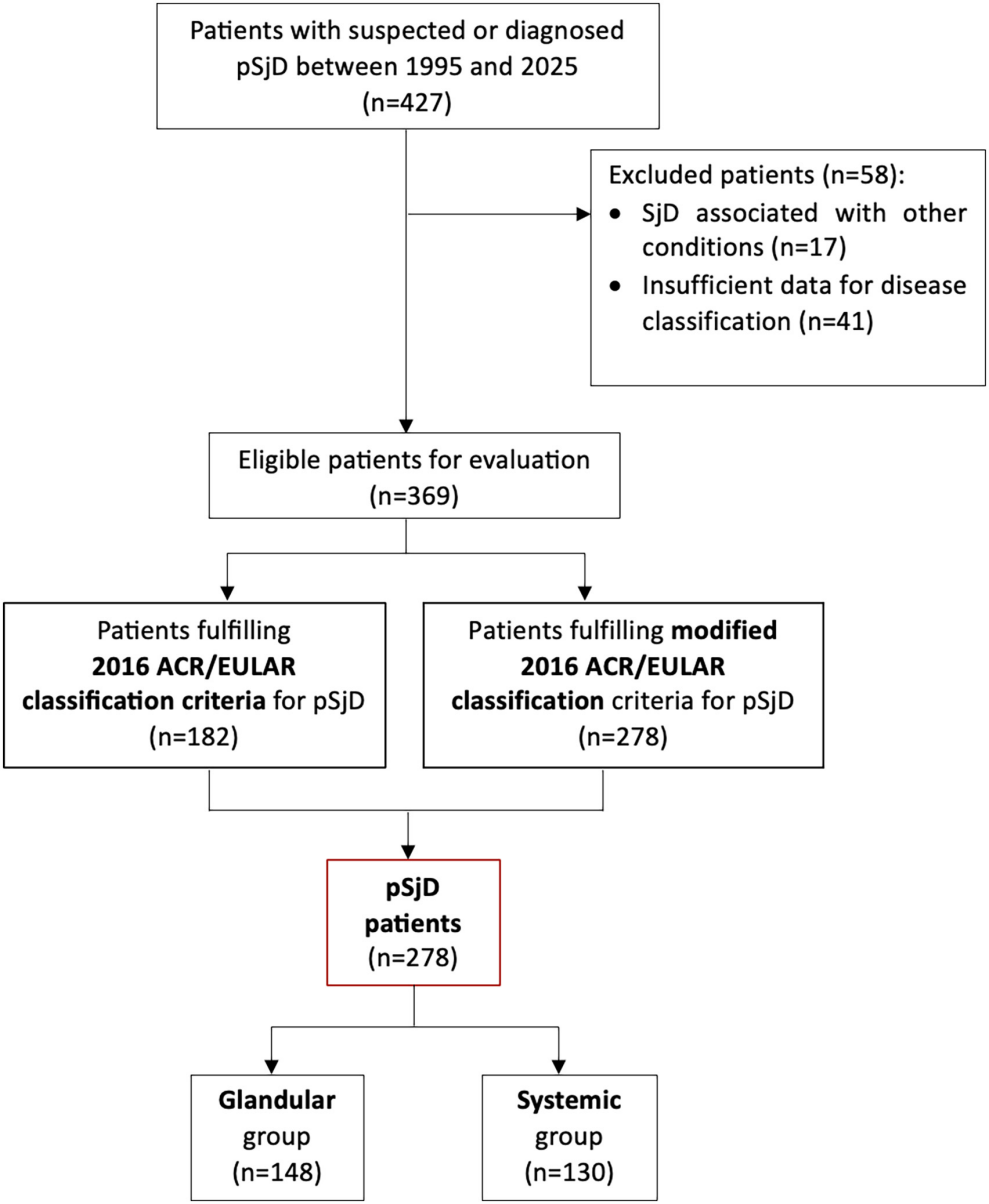
Flow diagram of patients according to the 2016 EULAR/ACR classification criteria and the modified 2016 EULAR/ACR criteria, with stratification into glandular and systemic clinical phenotype. pSjD, primary Sjögren disease.

A cohort of 427 patients with suspected or established pSjD evaluated between 1995 and 2025 was screened. Of these, 58 were excluded, including 17 with associated conditions and 41 with insufficient data to apply the classification criteria. After exclusions, 369 patients remained eligible for further evaluation. Among them, 182 fulfilled the 2016 ACR/EULAR classification criteria, whereas an additional 96 fulfilled only the modified criteria, resulting in a final cohort of 278 patients. Of these, 148 (53%) were classified as having a glandular phenotype and 130 (47%) as having a systemic phenotype (Figure 1). SGUS was available in 176 patients, with 115 examinations performed by EN and 61 by experienced radiologists at our center.

Baseline demographic and clinical characteristics were broadly comparable between phenotypic groups, including age at diagnosis, sex, ethnicity, sicca manifestations, and the frequency of abnormal salivary gland biopsy and pathological SGUS findings (Table 2). Disease activity was higher in patients with systemic involvement, who also showed a greater proportion of moderate-to-high ESSDAI scores (Table 2).

**TABLE 2:** Classification criteria, clinical characteristics and disease activity of included patients.

	Glandular (*n* = 148)	Systemic (*n* = 130)	Total (*n* = 278)	*P*-value
Demographic data				
Age at the time of study	60.2 ( ± 14.3)	62.7 ( ± 14.9)	61.3 ( ± 14.6)	0.15
Age at disease diagnosis	55.1 ( ± 14.0)	54.0 ( ± 15.2)	54.6 ( ± 14.5)	0.51
Ethnic origin				
Caucasian	117 (79)	113 (87)	230 (83)	0.34
Hispanic	25 (17)	15 (12)	40 (14)	
Asian	4 (3)	1 (1)	5 (2)	
African	2 (1)	1 (1)	3 (1)	
Women	140 (95)	125 (96)	265 (95)	0.54
Other autoimmune manifestations				
Primary biliary cholangitis	3 (2)	7 (5)	10 (4)	0.20
Autoimmune hepatitis	2 (1)	2 (2)	4 (1)	1.00
Classification criteria				
Positive anti-SSA/Ro	141 (95)	125 (96)	266 (96)	0.72
Positive glandular biopsy*	39/54(72)	36/44 (82)	75/98 (77)	0.22
Schirmer test (pathologic)	76/88 (86)	64/72 (89)	140/160 (88)	0.63
Pathologic ultrasound* (major salivary gland)	79/100 (79)	56/76 (74)	135/176 (77)	0.47
Clinical manifestations				
Xerophthalmia	135 (91)	112 (86)	247 (89)	0.18
Xerostomia	127 (86)	106 (82)	233 (84)	0.34
Renal involvement	0 (0)	6 (5)	6 (2)	0.01
Cytopenias	2 (1)	25 (19)	27 (10)	<0.001
Cutaneous manifestations	0 (0)	34 (26)	34 (12)	<0.001
Leukocytoclastic vasculitis	0 (0)	17 (13)	17 (6)	<0.001
Interstitial lung disease	0 (0)	21 (16)	21 (8)	<0.001
Myositis	0 (0)	2 (2)	2 (1)	0.22
Polyneuropathy	0 (0)	12 (9)	12 (4)	<0.001
Arthritis	0 (0)	49 (38)	49 (18)	<0.001
Parotidomegaly	6 (4)	15 (12)	21 (8)	0.02
Central nervous system	0 (0)	6 (5)	6 (2)	0.01
Asthenia	41 (28)	30 (23)	71 (26)	0.38
ESSDAI	4.0 ( ± 7.9)	13.9 ( ± 21.7)	8.6 ( ± 16.7)	< 0.001**
Low activity (<5)	116 (78)	68 (52)	184 (66)	
Moderate activity (5–13)	20 (14)	28 (22)	48 (17)	
High activity (≥14)	12 (8)	34 (26)	46 (17)	

*Data are shown as number of positive cases/number of patients tested.

** U Mann-Whitney test. Data are expressed as mean (SD) or n (%). ESSDAI: EULAR Sjögren’s Syndrome Disease Activity Index.

Serological and laboratory findings were also broadly similar across groups, including autoantibody profile, rheumatoid factor, antinuclear antibodies, immunoglobulin-related parameters, monoclonal gammopathy, and complement levels (Table 3).

**TABLE 3:** Immunological and laboratory findings of patients included in the assessment.

Immunological findings	Glandular (*n* = 148)	Systemic (*n* = 130)	Total (*n* = 278)	*P*-value
Anti Ro-52+	98 (66)	95 (73)	193 (69)	0.40
Anti Ro-60+	113 (76)	100 (77)	213 (77)	0.87
Anti La+	67 (45)	58 (45)	125 (45)	0.88
Rheumatoid factor +	82 (55)	76 (58)	158 (57)	0.61
ANA +	140 (95)	123 (95)	263 (95)	0.78
Laboratory findings				
Hypergammaglobulinemia	67 (46)	55 (43)	122 (45)	0.55
Increased β2-microglobulin	39 (26)	35 (27)	74 (27)	0.79
Monoclonal spike	20 (14)	19 (15)	39 (15)	0.80
MGUS	22 (15)	17 (13)	39 (14)	0.69
C3 (mg/dL)	117 ( ± 22)	118 ( ± 26)	118 ( ± 24)	0.75
C4 (mg/dL)	22 ( ± 8)	22 ( ± 12)	22 ( ± 10)	0.47
Total cholesterol (mg/dL)	197 ( ± 37)	188 ( ± 36)	193 ( ± 37)	0.05
HDL-cholesterol (mg/dL)	62 ( ± 16)	62 ( ± 18)	62 ( ± 17)	0.83
LDL-cholesterol (mg/dL)	118 ( ± 31)	109 ( ± 32)	113 ( ± 32)	0.05
Uric acid (mg/dL)	4.6 ( ± 1.4)	4.6 ( ± 1.3)	4.6 ( ± 1.3)	0.84
Serology HBV and/or HCV+	4 (3)	4 (4)	8 (4)	1.00
SGUS				
SGUS performed	103 (70)	71 (55)	174 (63)	0.014
Pathological SGUS	82/103(80)	52/71 (73)	134/174(77)	0.362

Data are expressed as mean (SD) or n (%). ANA, antinuclear antibodies; C3, complement component 3; C4, complement component 4; MGUS, monoclonal gammopathy of undetermined significance; HBV, hepatitis B virus; HCV, hepatitis C virus; HDL, high-density lipoprotein; LDL, low-density lipoprotein; SGUS, Salivary Gland Ultrasonography.

The main differences between phenotypes were observed in relation to neoplasia and treatment exposure. Patients with systemic involvement had a higher frequency of neoplasia both before and after pSjD diagnosis, particularly lymphoma, which was diagnosed in 15/278 patients (5%) overall and was significantly more frequent in the systemic group than in the glandular group (10% vs. 1%, *p* = 0.001) (Table 4). By logistic regression, we confirmed the association between lymphoma and glandular phenotype (crude OR = 8.11 [95% CI: 1.79, 36.7] and age and duration adjusted OR = 6.48 [95% CI: 1.40, 30.0]). By contrast, differences in solid tumors did not reach statistical significance (Table 4). 4/15 patients with lymphoma had been diagnosed before the diagnosis of pSjD. All these patients had systemic involvement. Lymphoma could be considered as the first manifestation of the disease in these patients.

**TABLE 4:** Occurrence of neoplasms in 278 Patients with pSS.

Neoplasia	Glandular (*n* = 148)	Systemic (*n* = 130)	Total (*n* = 278)	*P*-value
Before pSS diagnosis	4 (3)	14 (11)	18 (6)	0.007
After pSS diagnosis	9 (6)	18 (14)	27 (10)	0.03
Lymphoma	2 (1)	13 (10)	15 (5)	0.001
Non-Hodgkin lymphoma	2 (1)	11 (9)	13 (5)	0.008
Leukemia	0 (0)	1 (1)	1 (0)	0.46
Solid neoplasia	11 (7)	17 (13)	28 (10)	0.12

Data are expressed as n (%). pSS, Primary Sjögren Syndrome.

Patients with systemic involvement also more frequently received glucocorticoids and conventional immunosuppressants, while hydroxychloroquine use was comparable between groups (Table 5). Rituximab exposure was recorded only when prescribed for systemic manifestations and did not include administrations given for lymphoma treatment.

**TABLE 5:** Treatment exposure from included patients.

Treatment	Glandular (*n* = 148)	Systemic (*n* = 130)	Total (*n* = 278)	*P-*value
Hydroxychloroquine	33 (22)	31 (24)	64 (23)	0.76
Leflunomide	0 (0)	6 (5)	6 (2)	0.01
Azathioprine	0 (0)	10 (8)	10 (4)	<0.001
Methotrexate	0 (0)	12 (9)	12 (4)	<0.001
Mycophenolate	1 (1)	2 (2)	3 (1)	0.60
Intravenous immunoglobulins	0 (0)	2 (2)	2 (1)	0.22
Rituximab	3 (2)	12 (9)	15 (5)	0.01
Prednisone	9 (6)	29 (22)	38 (14)	<0.001

Data are expressed as n (%).

## 4 Discussion

In the present cohort, the systemic phenotype of pSjD was characterized by a wide spectrum of organ involvement, including musculoskeletal, hematological, pulmonary, neurological, and renal manifestations. This phenotype reflects a greater systemic disease burden and has been associated with less favorable outcomes in pSjD (33, 34). This observation is in line with data from large international cohorts highlighting the marked clinical heterogeneity of pSjD. In this regard, an artificial intelligence–driven analysis of more than 17,000 patients from the Sjögren Big Data Consortium identified distinct phenotypic patterns at diagnosis, with systemic manifestations clustering in subgroups characterized by higher disease burden, particularly among male patients (35).

These data further indicate that specific immunological signatures at diagnosis are strongly associated with higher systemic disease activity, as assessed by ESSDAI, supporting the clinical relevance of serological patterns for identifying patients at higher risk of systemic involvement in pSjD (36). In our series, systemic disease activity, measured by ESSDAI, was higher in patients with a systemic phenotype. Although ESSDAI is a validated and widely used index in pSjD (37), it reflects disease activity at the time of assessment, whereas the clinical phenotype used in this study was designed to capture the cumulative pattern of organ involvement over time. This distinction may be particularly important when assessing lymphoma risk, as lymphoma usually occurs later in the disease course and a cross-sectional activity measure may not fully represent cumulative disease burden. Thus, while disease activity has previously been explored in relation to lymphoma (38), ESSDAI alone may be insufficient in this context. In this regard, our findings support the use of clinical phenotype stratification as a complementary approach that may provide additional information when considering lymphoma risk (39) and it should be considered together with immunological and disease activity measures (7, 40).

In our cohort, incorporation of SGUS into the modified 2016 ACR/EULAR criteria may have facilitated the classification of additional 96 patients with pSjD who would not have fulfilled the original 2016 ACR/EULAR criteria, thereby reducing the number of patients that needed to undergo labial biopsy to be classified. This is consistent with previous studies showing that SGUS has a weight like other minor items, improves sensitivity, and may help classify a subset of patients without substantially altering overall diagnostic performance (21, 28–30).

In our pSjD cohort, neoplastic disease, mainly hematological, was more frequent among patients with systemic involvement, both before and after pSjD diagnosis. Most hematological malignancies were non-Hodgkin lymphomas, consistent with prior reports identifying lymphoproliferative disorders as a major disease-related complication in pSjD (41). Our findings align with large cohort and population-based studies showing a substantially increased lymphoma risk in pSjD (42). In contrast, evidence for an increased risk of non-lymphoid malignancies remains heterogeneous across studies (42, 43).

From a pathophysiological perspective, lymphomagenesis in pSjD is thought to be driven by chronic antigen-dependent B-cell stimulation, leading to clonal expansion and malignant transformation. The higher frequency of lymphoma observed in patients with systemic disease is consistent with the concept that immune stimulation and loss of B-cell tolerance may extend beyond the exocrine glands in a subset of patients with pSjD (33).

Several clinical and immunological features associated with B-cell hyperactivity have been proposed as predictors of lymphoma development in pSjD. Previous studies have highlighted persistent parotidomegaly, cytopenias, hypocomplementemia, cryoglobulinemia, and monoclonal gammopathy as key elements of the lymphoma-prone phenotype (44). In our cohort, several of these features, particularly parotidomegaly and hematological abnormalities, were more frequently observed in patients with systemic disease, in whom lymphoma was more common. These observations highlight the importance of detailed clinical characterization when assessing lymphoma risk in patients with pSjD. In this regard, previous studies suggest that baseline serological abnormalities alone may not adequately predict lymphoma risk and should be interpreted in conjunction with clinical manifestations and disease course (10).

Geographic variability has also been shown to influence the clinical and serological expression of pSjD. In a large Italian cohort, relevant differences in systemic manifestations and autoantibody profiles were observed across different macro-areas, highlighting the impact of population-related factors on disease phenotype (45). In regard to this, the monocentric design of our study allowed the analysis of a geographically homogeneous cohort assessed with a standardized clinical and immunological approach, thereby reducing inter-center variability.

Our present study has several limitations. Its retrospective design may have resulted in incomplete data capture and limited the ability to assess longitudinal changes in disease activity and immunological markers. The relatively small number of lymphoma cases did not allow multivariable analyses to identify independent predictors. Another limitation is that not all SGUS examinations were performed by the same operator. While examinations performed by EN were graded according to the OMERACT scoring system, those performed by radiologists were evaluated according to the reported glandular abnormalities. This may have introduced operator-dependent variability and potential misclassification.

Despite these limitations, the strengths of this study include a well-characterized homogeneous cohort with clearly defined phenotypic stratification based on a detailed evaluation of clinical, immunological, imaging, and disease activity parameters. Our findings support the concept that patients with pSjD and systemic involvement represent a subgroup at increased risk of lymphoma. Comprehensive clinical characterization and long-term follow-up may therefore help identify patients who could benefit from closer monitoring. Future prospective studies integrating longitudinal clinical data, biomarkers, and imaging are needed to better define lymphoma risk stratification in pSjD.

## Author’s note

This study was presented in part at the American College of Rheumatology (ACR) Convergence held in Chicago in 2025.

## Data Availability

The original contributions presented in this study are included in this article/supplementary material, further inquiries can be directed to the corresponding authors.
